# Serum-Mediated Oxidative Stress from Systemic Sclerosis Patients Affects Mesenchymal Stem Cell Function

**DOI:** 10.3389/fimmu.2017.00988

**Published:** 2017-09-01

**Authors:** Guillaume Fonteneau, Claire Bony, Radjiv Goulabchand, Alexandre T. J. Maria, Alain Le Quellec, Sophie Rivière, Christian Jorgensen, Philippe Guilpain, Danièle Noël

**Affiliations:** ^1^IRMB, INSERM, Montpellier University, Montpellier, France; ^2^Department of Internal Medicine, Multiorganic Diseases, Saint-Eloi Hospital, Montpellier, France; ^3^Clinical Immunology and Osteoarticular Diseases Therapeutic Unit, Lapeyronie Hospital, Montpellier, France

**Keywords:** mesenchymal stem cells, systemic sclerosis, cell therapy, oxidative stress, advanced oxidation protein product

## Abstract

**Objectives:**

Properties of mesenchymal stromal/stem cells (MSCs) from systemic sclerosis (SSc) patients have been reported to be altered. MSC-based therapy may therefore rely on the use of allogeneic MSCs from healthy subjects. Here, we investigated whether heterologous MSCs could exhibit altered properties following exposure to oxidative environment of SSc sera.

**Methods:**

Human bone marrow-derived MSCs were cultured in the presence of various sera: control human serum AB (SAB), SAB with HOCl-induced AOPPs at 400 or 1,000 µmol/L (SAB_400_ or SAB_1000_, respectively), or H_2_O_2_-induced AOPPs or SSc patient serum (PS). Proliferation, apoptosis, and senescence rates of MSCs were evaluated after 3, 6, and 10 days in culture. Reactive oxygen species and nitric oxide production were quantified at 24 h. Trilineage potential of differentiation was tested after 21 days in specific culture conditions and immunosuppressive function measured in a T lymphocyte proliferative assay.

**Results:**

In the presence of oxidative environment of PS, MSCs retained their proliferative potential and survived for at least the first 3 days of exposure, while the number of senescent MSCs increased at day 6 and apoptosis rate at day 10. Exposure to PS enhanced the antioxidant capacity of MSCs, notably the expression of SOD2 antioxidant gene. By contrast, the osteoblastic/adipogenic potential of MSCs was increased, whereas their immunosuppressive function was slightly reduced.

**Discussion:**

Although some functional properties of MSCs were affected upon culture with PS, evidence from preclinical studies and the present one suggested that MSCs can adapt to the oxidative environment and exert their therapeutic effect.

## Introduction

Systemic sclerosis (SSc) (also called scleroderma) is a rare autoimmune disorder characterized by multiorgan fibrosis, vascular involvement, and production of autoantibodies. SSc exhibits a severe prognosis associated with specific organ involvements and premature mortality and can still be considered as an intractable disease. Oxidative stress plays a crucial role in the development of SSc, leading to fibroblast proliferation and endothelial cell apoptosis. This role is illustrated by the link between environmental exposure to oxidants and professional disease (1) and also by the increased levels of oxidative markers [such as advanced oxidation protein products (AOPP)] observed in serum of SSc patients (2). Serum of SSc patients with diffuse cutaneous forms contained AOPP concentrations of 400 µmol/L of chloramine-T equivalents, while those levels are around 200 µmol/L of chloramine-T equivalents in limited cutaneous forms of SSc and healthy individuals (3). AOPPs are the results of protein oxidation by either hypochlorous acid (HOCl) or hydrogen peroxide (H_2_O_2_) and appear to contribute to disease pathophysiology. In addition, SSc serum can induce the production of different types of reactive nitric species (RNS) and reactive oxygen species (ROS), selectively activating endothelial cells or fibroblasts (2). Thus, SSc serum represents an oxidative environment specific to the disease that can also trigger amplification loop leading to vasculopathy and fibrosis.

Because of their trophic and immunomodulatory properties, mesenchymal stromal/stem cells (MSCs) represent one of the most promising therapeutic approaches in SSc. MSCs are non-hematopoietic multipotent progenitor cells, which can be isolated from bone marrow, adipose tissue, or umbilical cord, and exhibit a tripotential of differentiation toward adipocytes, chondrocytes, and osteoblasts (4). They have proven efficacy in several animal models of fibrosis and in the murine model of HOCl-induced SSc (5). In addition, the therapeutic interest of allogeneic MSCs is being evaluated in SSc patients in phase I/II studies (NCT00962923 and NCT02213705).

Noteworthy, allogeneic MSC-based therapy could appear safer than autologous approach in SSc. Indeed, SSc-MSCs were shown to display altered phenotypical and functional properties. They expressed higher levels of transforming growth factor (TGF)-βRII (6), α-smooth muscle actin, SM22α genes (7), and senescence markers (8) as well as enhanced pro-angiogenic activity (9). Since resident MSCs obtained from SSc patients (SSc-MSCs) may present altered functions, use of allogeneic MSCs could be more appropriate in the clinics and has already shown some promising results (10). Yet, the possibility that heterologous MSCs could exhibit altered properties following exposure to SSc oxidative environment has not been investigated. Here, we evaluated whether oxidative stress, and particularly HOCl and induced AOPPs, might affect the characteristics and functional properties of heterologous human MSCs when exposed to serum from SSc patients.

## Materials and Methods

### Human Sample Collection

Seventeen patients were included for serum collection. Their clinical and biological characteristics are indicated in Table 1. Human bone marrow-derived MSCs were isolated from patients undergoing hip replacement surgery as described earlier (11). Both human samples were obtained from patients with written informed consent from all subjects in accordance with the Declaration of Helsinki. This study was carried out in accordance with the recommendations of Committee for Person Protection of Languedoc-Roussillon and approved by the French Ministry of Higher Education and Research (DC-2010-1185 for MSC and DC-2014-2328 for SSc serum). MSCs were characterized by phenotyping and trilineage differentiation potential as described in Ref. (12) and used before passage 5. They were maintained in proliferative medium consisting in α-MEM (Lonza), 1 ng/mL of basic fibroblast growth factor (R&D Systems), 100 µg/mL penicillin/streptomycin (Lonza), 2 mM glutamine (Lonza), and supplemented with 10% fetal calf serum (FCS) before use in experimental settings. Blood from SSc patients was centrifuged at 2,000 *g* for 15 min, and patient serum (PS) stored at −80°C. Serum AB (SAB) was a pool of 200 human male AB plasma purchased from Sigma-Aldrich (ref H4522). Human blood was purchased from the Etablissement Français du Sang (Toulouse). Human peripheral blood mononuclear cells (PBMCs) were isolated by Ficoll (GE Healthcare) according to standard procedures.

**Table 1:** Clinical characteristics of SSc patients.

Patient (*N*)	Age at diagnosis (years)/sex	Disease duration from diagnosis (years)	Type of SSc	Autoantibodies	Clinical involvement	Immunosuppressive drugs at sampling time	Other treatments	AOPP plasma level (chloramine-T equivalents, μmol/L)
1	54/F	16	d-SSc	Scl70	CIPO, DU, GER, ILD, PAH	Low-dose steroids, azathioprine	Bosentan^a^, tadalafil^a^, low-dose steroids, cyclophosphamide, MMF, sildenafil, treprostinil, tadalafil, iloprost	121
2	27/F	16	d-SSc	Scl70, ANA	DU, GER, ILD	None	Bosentan^a^, sildenafil^a^	253
3	61/F	2	lc-SSc	Cm, PM/Scl	GER	None	None	267
4	32/M	25	d-SSc	Scl70, PM/Scl, ANA	CIPO, DU, GER, ILD, PAH, My	MMF	D-penicillamine	269
5	49/F	14	lc-SSc	ANA	GER, SRC	None	None	293
6	21/F	49	lc-SSc	Scl70, ANA	DU, GER, ILD	None	None	302
7	49/F	10	d-SSc	Scl 70	J, CIPO, DU, GER, ILD, PAH, SC	Low-dose steroids	Bosentan^a^, azathioprine, MTX, MMF, iloprost	319
8	65/F	0	lc-SSc	Scl70, ANA	GER, ILD, PAH	None	None	378
9	31/F	27	d-SSc	Scl70, SSa	DU, GER, ILD, PAH	MMF	Bosentan^a^, sildenafil^a^, iloprost^a^	390
10	21/F	1	lc-SSc	ANA	DU, GER, ILD	None	Low-dose steroids	394
11	22/F	7	lc-SSc	ANA	DU, GER, My	IvIg	None	508
12	57/F	16	lc-SSc	Scl70, ANA	DU, GER, ILD	None	Bosentan, iloprost	553
13	81/F	3	lc-SSc	ANA	My, PAH	Low-dose steroids, IvIg	None	575
14	52/F	17	d-SSc	Scl70, ANA	J, DU, GER, ILD, SC	None	Bosentan	613
15	46/F	29	lc-SSc	Cm, ANA, CCP	GER, SC	Low-dose steroids, MTX	Leflunomide, abatacept	677
16	47/F	8	d-SSc	ANA	J, DU, GER, SC, SRC	None	None	886
17	49/F	6	lc-SSc	Cm, PM/Scl	DU, GER, SC	None	Bosentan^a^	1,248

*All patients presented with Raynaud phenomenon and were treated with symptomatic treatments (calcium channel blockers, proton pump inhibitors)*.

*ANA, antinuclear antibody; AOPP, advanced oxidation protein products; CCP, anticyclic citrullinated peptide; CIPO, chronic intestinal pseudo-obstruction; Cm, anticentromere antibody; d-SSc, diffuse systemic sclerosis; DU, digital ulcers; F, female; GER, gastroesophageal reflux; ILD, interstitial lung disease; IvIg, intravenous immunoglobulins; J, joints; lc-SSc, limited cutaneous systemic sclerosis; M, male; MMF, mycophenolate mofetil; MTX, methotrexate; My, myositis; PAH, pulmonary arterial hypertension; Scl70, anti-Scl70 antibody; SC, subcutaneous calcinosis; SRC, scleroderma renal crisis; SSa, anti-Ro/SSa antibody; SSc, systemic sclerosis*.

*^a^Other treatment at sampling time*.

### Oxidative Medium Preparation

Healthy SAB (15 mL) was oxidized with 15 mL hypochlorite (HOCl) at room temperature for 1 h. HOCl was produced by adding 166 µL of NaClO solution to 11.1 mL of KH_2_PO_4_ solution (100 mM, pH 7.2), and HOCl concentration was determined by spectrophotometry at 292 nm as described earlier (2). Oxidized serum was then dialyzed in PBS at 4°C overnight using a semipermeable membrane (3.5 K molecular weight cut-off; Slide-A-Lyzer dialysis cassette, ThermoFisher Scientific). The AOPP level in oxidized SAB and PS was determined by spectrophotometry as previously described (2) and expressed as chloramine-T equivalents (μmol/L). Oxidized SAB was diluted with SAB to obtain a defined AOPP level of 400 µmol/L (SAB_400_) or 1,000 µmol/L (SAB_1000_). As control, H_2_O_2_ was added in culture medium with SAB (SAB_H2O2_) at 150 µM final concentration, which corresponded to 680 µmol/L of AOPPs.

### Proliferation Assay

MSCs were plated at 10,000 cells/cm^2^ in six-wells plates in proliferative medium supplemented with 5% human serum (SAB, SAB_400_, SAB_1000_, SAB_H2O2_, or PS). Media were changed every 3 days, and viable cells were counted using a Malassez hemocytometer at 3, 6, and 10 days. Results were expressed as the percentage of proliferation ± SEM and normalized at 100% for initially plated cell number.

### Apoptosis Assay

MSCs were plated at 10,000 cells/cm^2^ in six-well plates in proliferative medium containing 5% human serum (SAB, SAB_400_, SAB_1000_, SAB_H2O2_, or PS). Number of apoptotic cells was evaluated by Annexin V and 7-AAD labeling. Briefly, 10^5^ MSCs were suspended in 300 µL Annexin V binding buffer (BD Biosciences) and incubated with 2.5 µL of fluorescein isothiocyanate-conjugated Annexin V and 7-AAD antibodies (BD Biosciences) for 15 min at room temperature. The labeled cells were analyzed using a FACSCanto cytometer and the BD FACSDiva™ software V.6.1.3 (BD Biosciences). Results were expressed as the percentage of Annexin V^+^ and 7-AAD^−^ cells or gene expression fold change ± SEM and normalized to 1 for MSCs cultured in control SAB.

### Senescence Assay

MSCs were plated at 8,000 cells/cm^2^ in 12-well plates for senescence-associated β-galactosidase (SA-β-gal) staining or 10,000 cells/cm^2^ in 6-well plates for quantitative assay and cultured in proliferative medium containing 5% human serum (SAB, SAB_400_, SAB_1000_, SAB_H2O2_, or PS). For senescence-associated β-galactosidase (SA-β-gal) staining, cells were fixed with 2.5% glutaraldehyde for 10 min and incubated in staining solution at 37°C overnight. Staining solution consisted of 5 mM potassium ferrocyanide, 5 mM potassium ferricyanide, 1 mg/mL Xgal (Promega), 40 nM citric acid/sodium phosphate (pH 6), 0.15 M NaCl, 20 mM MgCl_2_, and pH was adjusted between 5.9 and 6.1. For quantitative evaluation of senescence, 15,000 cells were assayed using the Quantitative Cellular Senescence Assay Kit (Cells Biolabs, Clinisciences). Results were expressed as the relative fluorescence unit (RFU) or gene expression fold change ± SEM and normalized to 1 for MSCs cultured in control SAB.

### ROS Quantification

Quantification of ROS and nitric oxide (NO) production was performed using 6-carboxy-2′,7′-dichlorodihydrofluorescein diacetate (DCFDA; ref C2938) and 4-amino-5-methylamino-2′,7′-difluorofluorescein diacetate (DAF-FM; ref D23844) probes, respectively, following supplier’s recommendations (Molecular Probes, ThermoFisher Scientific). Briefly, MSCs were plated at 50,000/cm^2^ in six-well plates in proliferative medium and incubated with 50 µM of DCFDA or 10 µM of DAF2 at 37°C for 60 min. Probes were then washed out with PBS. Media containing 5% human serum (SAB, SAB_400_, SAB_1000_, SAB_H2O2_, or PS) were added for 24 h. Fluorescence was read at different time points using a Varioskan fluorometer (ThermoFisher Scientific) at 495 nm for excitation and 515 nm emission. Results were expressed as the relative fluorescence unit (RFU) or gene expression fold change ± SEM and normalized to 1 for MSCs cultured in control SAB.

### Differentiation Assays

#### Adipogenesis

MSCs were plated at 9,000 cells/cm^2^ in six-well plates and cultured in proliferative medium containing 10% FCS for 5 days. Differentiation media were DMEM-F12 (Lonza) with 100 µg/mL penicillin/streptomycin, 16 µM biotin, 18 µM panthotenic acid, 100 µM ascorbic acid, 60 µM indomethacin, 450 µM isobutylmethylxanthine, 1 µM dexamethasone, 1 µM rosiglitazone, and 5% human serum (SAB, SAB_400_, SAB_1000_, SAB_H2O2_, or PS). At day 21, cells were lyzed in RLT buffer for RT-qPCR or fixed with 2.5% glutaraldehyde, and lipid vacuoles were stained using Oil red O.

#### Chondrogenesis

Chondrogenic differentiation of MSCs was induced by 21-day culture in micropellets. Briefly, 250,000 cells were pelleted by centrifugation in 15-mL conical tubes and cultured in DMEM (Lonza) supplemented with 100 µg/mL penicillin/streptomycin, 0.35 mM proline, 0.1 µM dexamethasone, 0.17 mM ascorbic acid-2-phosphate, 1 mM pyruvate sodium, 1% insulin-transferrin-selenic acid (ITS) (Lonza), 10 ng/mL TGF-β3 (R&D Systems), and 5% human serum (SAB, SAB_400_, SAB_1000_, SAB_H2O2_, or PS). At day 21, micropellets were lyzed in RLT buffer and stored at −80°C.

#### Osteogenesis

MSCs were plated at 3,000 cells/cm^2^ in six-well plates and cultured in differentiation medium: DMEM (Lonza) with 10 mM β-glycerophosphate, 0.1 µM dexamethasone, 70 µM ascorbic acid-2-phosphate, 100 µg/mL penicillin/streptomycin, and 5% human serum (SAB, SAB_400_, SAB_1000_, SAB_H2O2_, or PS). At day 21, cells were lyzed in RLT buffer and stored at −80°C or fixed with 95% ethanol, and mineralization was observed after Alizarin Red S staining.

### RNA Extraction and RT-qPCR

RNA was extracted using the RNeasy mini kit (Qiagen) following the supplier’s recommendations. RNA (400 ng) was reverse transcribed using the Moloney Murine Leukaemia Virus Reverse Transcriptase (M-MLV) enzyme (Invitrogen, ThermoFisher Scientific). qPCR was then performed on 20 ng of cDNA using SybrGreen^®^ PCR Master Mix (Roche) with specific primers (Table S1 in Supplementary Material). PCR reaction was performed as follows: 95°C for 5 min; 40 cycles at 95°C for 15 s; 64°C for 10 s, and 72°C for 20 s in a LightCycler 480 instrument (Roche Diagnostics) or ViiA™ 7 Real-Time PCR System (Applied Biosystems, ThermoFisher Scientific). All values were normalized on RPS9 housekeeping gene and expressed as fold change using the formulae 2^−ΔΔCt^.

### T Lymphocyte Proliferative Assay

MSCs were cultured in proliferative medium containing 5% human serum (SAB, SAB_400_, SAB_1000_, SAB_H2O2_, or PS) for 3 days. They were then trypsinized and plated with 2 × 10^5^ PBMC at different densities to get ratios of 1/5, 1/10, and 1/50 (MSC/PBMC) in 96-well plates. Cells were cultured in IMDM (Invitrogen) containing 10% heat inactivated FCS, 100 µg/mL penicillin/streptomycin, 2 mM glutamine, 0.1 mM non-essential amino acids, 5 × 10^5^ M 2-mercaptoethanol, 1 mM sodium pyruvate, 10% FCS, 25 mM HEPES, and 2.5 µg/mL phytohemaglutinin (Sigma) for T lymphocyte activation. After 3 days, T lymphocyte proliferation was measured with Cell Proliferation ELISA, BrdU assay (Sigma-Aldrich). Results were expressed as the percentage of proliferation ± SEM and normalized at 100% for proliferation of activated PBMC minus basal proliferation.

### Statistical Analyses

Statistical analysis was performed with GraphPad 6 Prism Software. Data were compared using the Mann–Whitney test for non-parametric values. A *p* value <0.05 was considered significant.

## Results

### High AOPP Levels in SSc Patient Serum Affected the Proliferation Rate of MSC

**Figure 1:**
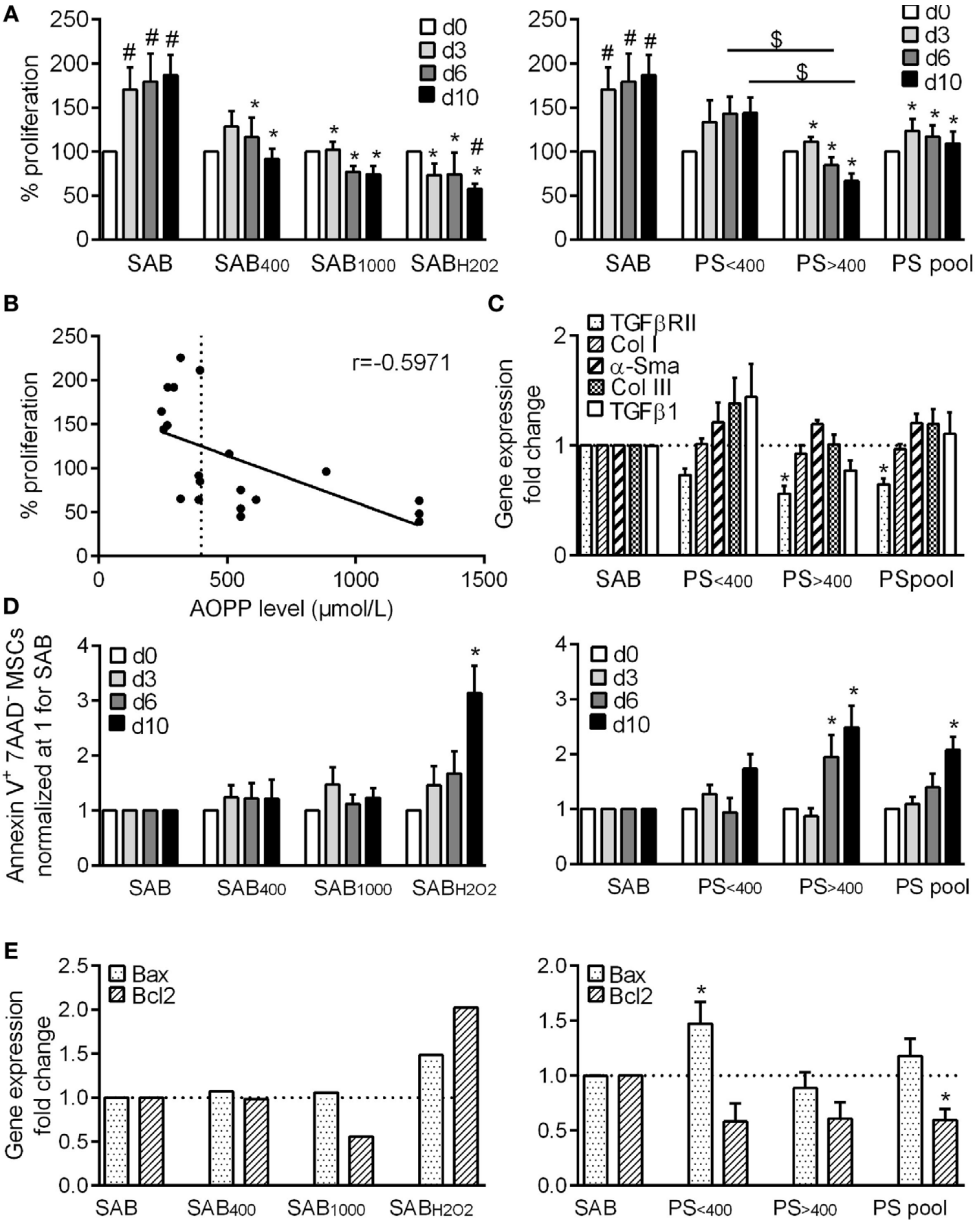
HOCl- or H_2_O_2_-induced serum AOPPs and systemic sclerosis (SSc) patient serum decreased the proliferation rate of MSCs and increased the number of apoptotic MSCs. **(A)** Percentage of MSC proliferation depending on the concentration of advanced oxidation protein products (AOPP) induced by HOCl in human serum AB (SAB): 400 µmol/L (SAB_400_) or 1,000 µmol/L (SAB_10000_), H_2_O_2_ (SAB_H2O2_) (*n* = 8), or SSc patient serum at different time points: day 3, 6, and 10. Patient serum (PS) were divided in two groups depending on AOPP levels: <400 μmol/L (PS_<400_; *n* = 11) or >400 μmol/L (PS_>400_; *n* = 9) or pooled in a single group (PS_pool_; *n* = 20). Data were normalized to 100% of cells plated at day 0. **(B)** Linear regression curve between percentage of MSC proliferation and AOPP level in SSc patient serum at day 10 (*n* = 20). *r* indicated the Pearson’s correlation coefficient. **(C)** Gene expression fold change of different profibrotic markers (PS_<400_ and PS_>400_, *n* = 4; PS_pool_, *n* = 8). **(D)** Percentage of Annexin V^+^ 7-AAD^-^ apoptotic MSCs at different time points (*n* = 8). **(E)** Gene expression fold change of proapoptotic marker Bax or antiapoptotic marker Bcl2 (PS_<400_ and PS_>400_, *n* = 4; PS_pool_, *n* = 8). Data were normalized to 1 for MSCs in SAB-containing medium. #*p* < 0.05 versus day 0; *p < 0.05 versus SAB at same time point; ^$^*p* < 0.05 versus indicated condition.

We first evaluated the proliferation rate of MSCs cultured for 10 days in medium containing 5% serum from healthy patients (SAB) or oxidized SAB that has been submitted to H_2_O_2_ (SAB_H2O2_) or HOCl treatment to get 400 or 1,000 µmol/L of AOPP (SAB_400_ and SAB_1000_). In SAB, MSCs rapidly proliferated during the first 3 days and then weakly proliferated till day 10 (Figure 1A). By contrast, MSCs cultured in oxidized SAB did not proliferate compared to day 0 and even died when cultured in SAB_H2O2_. By comparison with MSCs cultured in SAB, growth of MSCs in oxidized SAB was significantly inhibited at whatever the time point, indicating that oxidized human serum inhibited MSC proliferation.

We also investigated the growth rate of MSCs cultured with SSc patient serum (PS). MSCs cultured with PS (PS_pool_) did not proliferate compared to day 0, and proliferation was significantly inhibited compared to MSC in SAB-containing medium at each time point (Figure 1A). However, we noticed some differences according to the level of AOPP measured in SSc serum and segregated the data in two groups: serum with AOPP levels less than or more than 400 µmol/L (PS_<400_ or PS_>400_, respectively). The median AOPP level in PS was 390 µmol/L. Proliferation of MSCs cultured with PS_<400_ was not different from MSCs cultured with SAB, whereas proliferation of MSCs cultured with PS_>400_ was significantly inhibited and lower than that of MSCs cultured with PS_<400_ at all time points. Indeed, a negative linear correlation between the proliferation rate of MSCs and AOPP levels was found with a significant Pearson’s correlation coefficient *r* of −0.5971 (*p* = 0.0054) (Figure 1B). This could not be attributed to variability between MSC samples because proliferation rates of different MSC samples cultured with same patient serum were similar (data not shown). Finally, expression levels of five genes associated with SSc phenotype did not change (Figure 1C). Indeed, long-term exposure to high levels of AOPP and oxidative stress inhibited MSC proliferation but did not change the phenotype of MSCs.

### High AOPP Levels in Patient Serum Induced MSC Apoptosis

We also found that the percentage of apoptotic MSCs in control SAB was 5.33 ± 1.60% at day 3, 7.34 ± 1.77% at day 6, and 5.19 ± 1.17% at day 10. By comparison, no increase of apoptosis was noticed when MSCs were cultured with SAB_400_, SAB_1000_, or SAB_H2O2_ at day 3 or 6 (Figure 1D). At day 10, the percentage of apoptotic MSCs was significantly increased with SAB_H2O2_, which mirrored the lower proliferation rate of MSCs observed in Figure 1A. When MSCs were cultured with PS, the percentage of apoptotic cells was significantly increased at days 6 and 10 for MSCs expanded with PS_>400_ and PS_pool_ (Figure 1D). Expression levels of the pro-apoptotic marker Bax was increased with PS_<400_, and the antiapoptotic marker Bcl2 was significantly decreased (Figure 1E). Culture of MSCs with H_2_O_2_ or PS therefore induced a slight increase of apoptosis on the long term.

### Low AOPP Levels in Patient Serum Induced MSC Senescence

**Figure 2:**
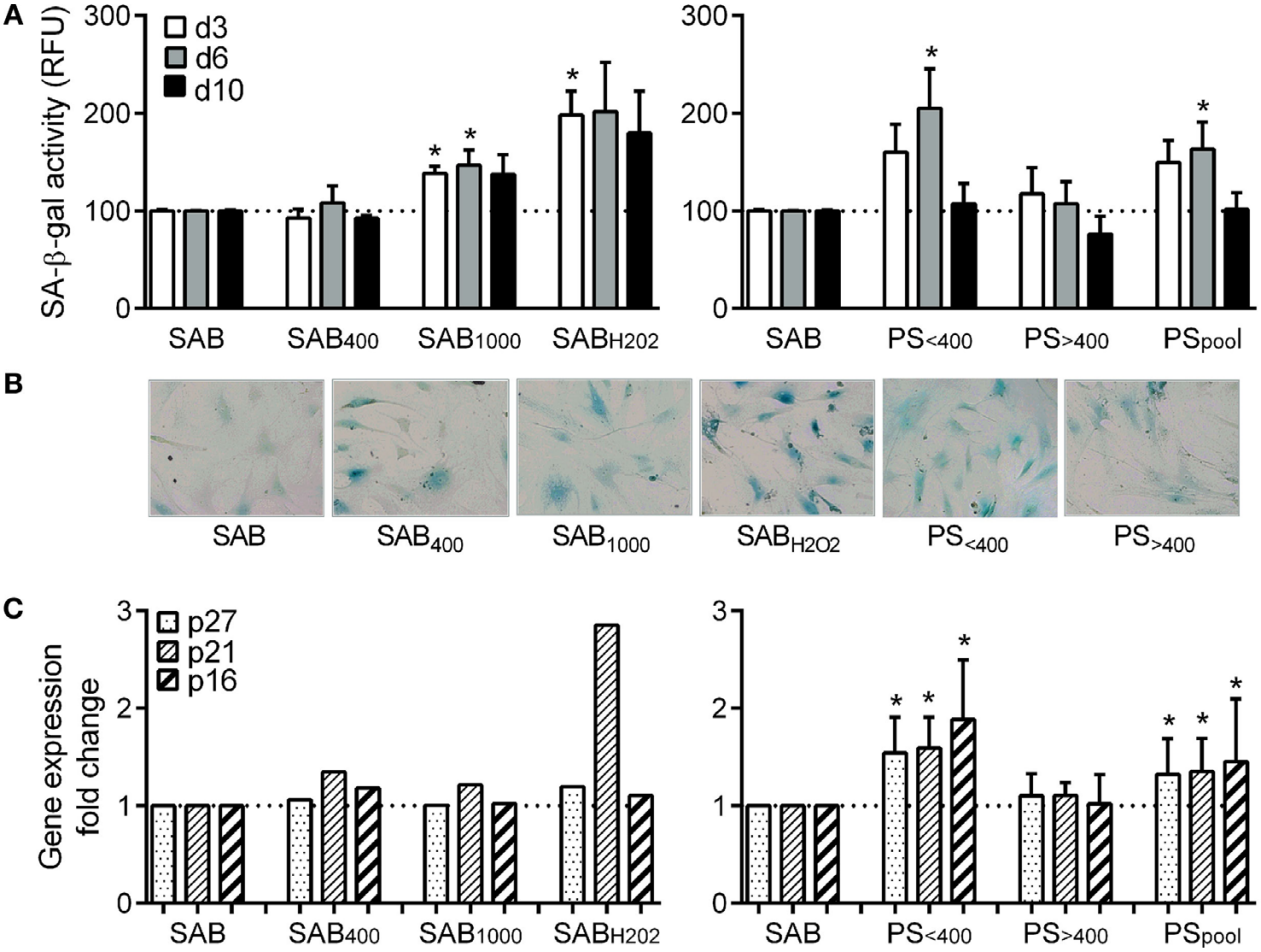
HOCl- or H_2_O_2_-induced serum AOPPs and systemic sclerosis (SSc) patient serum increased the number of senescent MSCs. **(A)** quantification of SA-β-gal activity in senescent MSCs in culture with human serum AB (SAB) or oxidized SAB (SAB_400_, SAB_1000_, and SAB_H2O2_) or SSc patient serum at different time points: day 3, 6, or 10 (*n* = 8). Senescence was measured using the quantitative cellular senescence assay kit (Cells Biolabs) and expressed as relative fluorescence unit (RFU). Sera from patient (PS) were divided in two groups depending on AOPP levels: <400 μmol/L (PS_<400_; *n* = 11) or >400 μmol/L (PS_>400_; *n* = 9) or pooled in a single group (PS_pool_; *n* = 20). Data were normalized to 100 for senescent MSCs detected in SAB-containing medium. **(B)** Representative photographs of SA-β-gal staining of MSCs cultured in same conditions as in **(A)**, at day 6. **(C)** Gene expression fold change of different senescence markers: p16, p21, and p27 in same conditions as in **(A)** (PS_<400_ and PS_>400_, *n* = 4; PS_pool_, *n* = 8). Data were normalized to 1 for apoptotic MSCs detected in SAB-containing medium. *p < 0.05 versus SAB at same time point.

We also assessed senescence by quantifying SA-β-gal activity of MSCs. In the SAB culture condition, we measured 143.7 ± 20.4 at day 3, 199.3 ± 23 at day 6, and 258 ± 80 RFU at day 10 that was normalized to 100 at each time point. Compared to SAB, SA-β-gal activity of MSCs was increased when cultured with SAB_1000_ or SAB_H2O2_ although significance was reached only at day 3 (Figure 2A). A significant increase of SA-β-gal activity was observed only for MSCs cultured with PS_<400_ and PS_pool_ at day 6. Qualitative assessment by SA-β-gal staining reflected quantitative analysis (Figure 2B). Expression levels of the senescent markers p16, p21, and p27 were increased when MSCs were cultured with PS_<400_ and PS_pool_ (Figure 2C). Altogether, the data pointed out induction of senescence in MSCs by oxidative stress (SAB_1000_ or H_2_O_2_) and PS_<400_.

### Patient Serum Did Not Induce Oxidative Stress in MSCs

**Figure 3:**
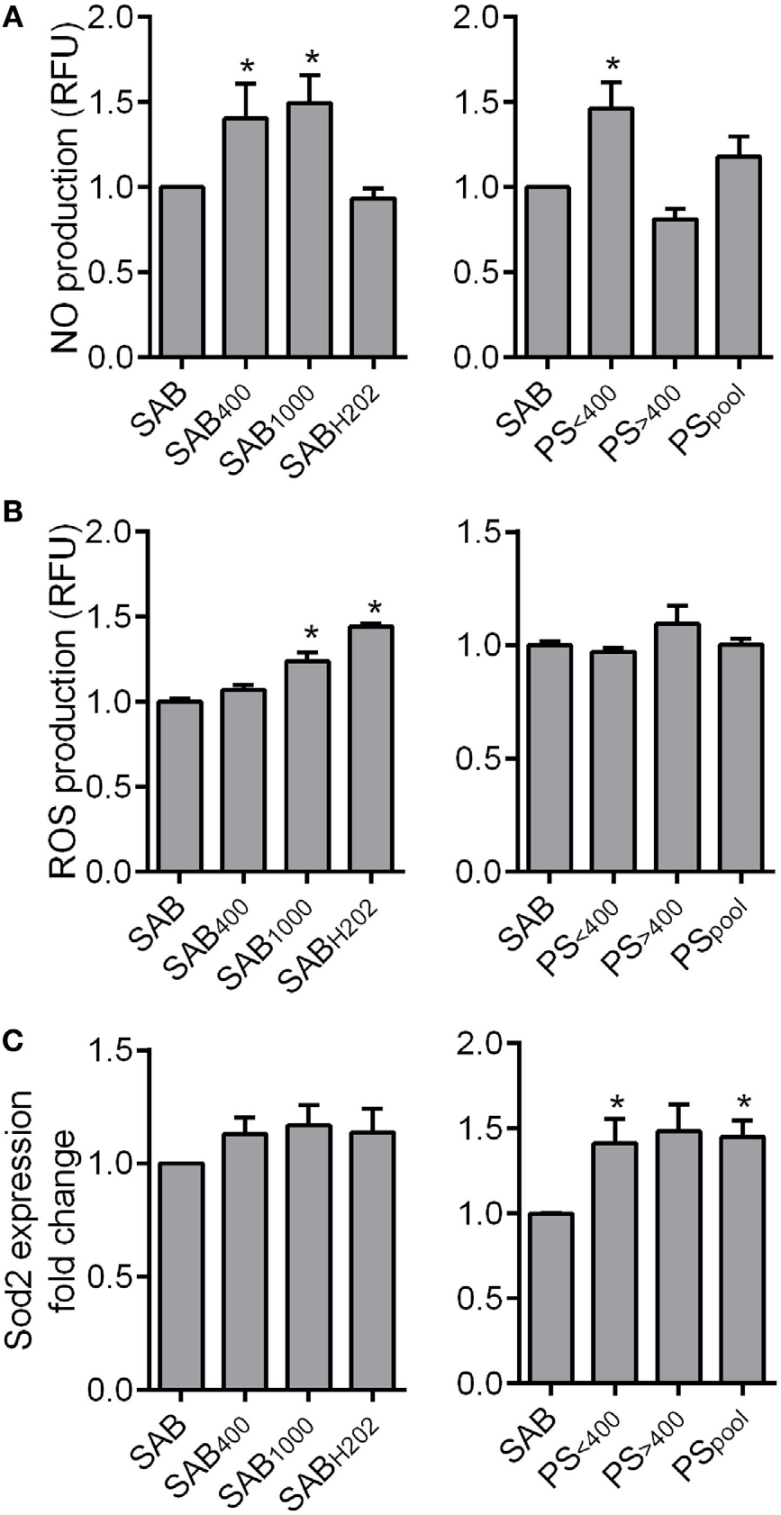
HOCl- or H_2_O_2_-induced serum AOPPs increased nitric oxide (NO) and reactive oxygen species (ROS) production in MSCs. **(A)** Quantification of NO production in MSCs in culture with human serum AB (SAB) or oxidized SAB (SAB_400_, SAB_1000_, and SAB_H2O2_; *n* = 7 for each condition) or SSc patient serum at 24 h. Sera from patient (PS) were divided in two groups depending on AOPP levels: <400 μmol/L (PS_<400_; *n* = 9) or >400 μmol/L (PS_>400_; *n* = 7) or pooled in a single group (PS_pool_; *n* = 16). NO production was measured using 4-amino-5-methylamino-2′,7′-difluorofluorescein diacetate probes (Molecular Probes, ThermoFisher Scientific). **(B)** Quantification of ROS production in MSCs in same conditions as in **(A)**. ROS production was measured using 6-carboxy-2′,7′-dichlorodihydrofluorescein diacetate probes (Molecular Probes, ThermoFisher Scientific). **(C)** Gene expression fold change of Sod2 antioxidant marker in same conditions as in **(A)** (PS_<400_ and PS_>400_, *n* = 4; PS_pool_, *n* = 8). Data were normalized to 1 for MSCs in SAB-containing medium. *p < 0.05 versus SAB at same time point.

We next measured the production of reactive oxygen and nitrogen species (ROS and RNS, respectively). MSCs cultured with SAB_400_, SAB_1000_, or PS_<400_ produced higher levels of NO than MSCs in SAB (Figure 3A), whereas PS_>400_ and PS_Pool_ did not significantly modify NO secretion. When looking at ROS production, SAB_1000_ and SAB_H2O2_ induced a significant increase of ROS production by MSCs, while PS did not alter ROS production (Figure 3B). We also noticed that the superoxide dismutase (Sod)2 antioxidant gene was increased in MSCs cultured with PS, whatever the AOPP levels, correlating the absence of ROS production (Figure 3C). Indeed, PS did not influence the production of RNS or ROS by MSCs but significantly increased expression of Sod2, suggesting a possible induction of antioxidative activity.

### Patient Serum Increased the Differentiation Capacities of MSCs toward Adipocytes and Osteoblasts

**Figure 4:**
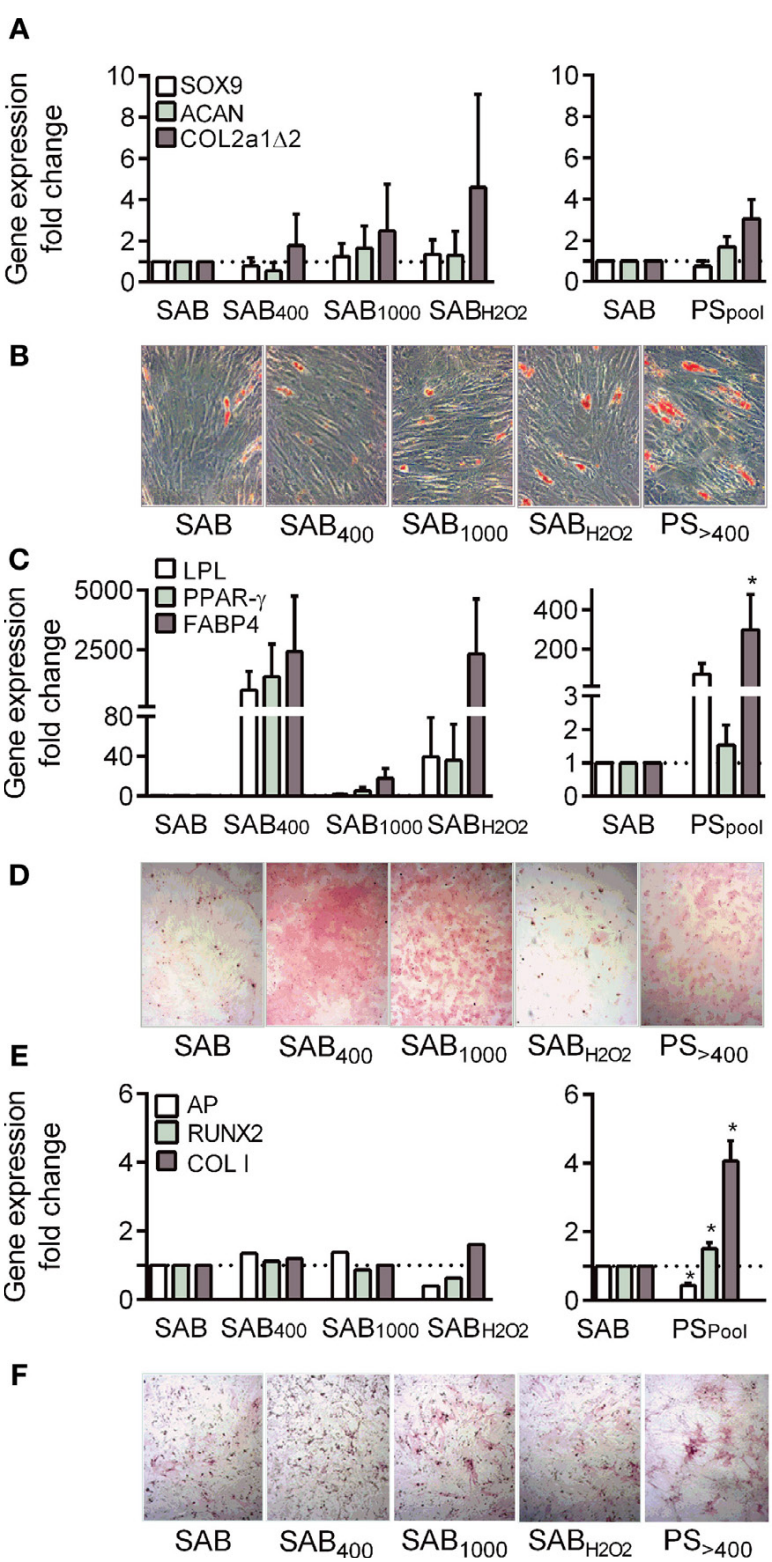
Systemic sclerosis (SSc) patient serum increased osteogenic differentiation potential of MSCs. **(A)** Chondrogenic differentiation of MSCs cultured with human serum AB (SAB) or oxidized SAB (SAB_400_, SAB_1000_, and SAB_H2O2_; *n* = 3 for each condition) or SSc patient serum (*n* = 13) at day 21. Chondrogenesis was assessed by measuring expression level of specific genes: sex-determining region Y-box9 (SOX9), aggrecan (ACAN), and type II collagen variant B (COL2a1Δ2) by RT-qPCR. **(B)** Representative photographs of MSCs depicted in **(C)** and stained with Oil Red O. **(C)** Adipogenic differentiation of MSCs cultured with human SAB or oxidized SAB (SAB_400_, SAB_1000_, and SAB_H2O2_; *n* = 3 for each condition) or SSc patient serum (*n* = 13) at day 21. Adipogenesis was assessed by measuring expression level of specific genes: lipoprotein lipase (LPL), peroxisome proliferator-activator receptor (PPAR)-γ, and fatty acid binding protein (FABP)4 by RT-qPCR. **(D)** Representative photographs of MSCs depicted in **(E)** and stained with Alizarin Red S. **(E)** Osteogenic differentiation of MSCs cultured with human SAB or oxidized SAB (SAB_400_, SAB_1000_, and SAB_H2O2_; *n* = 3 for each condition) or SSc patient serum (*n* = 13) at day 21. Osteogenesis was assessed by measuring expression level of specific genes: Runt-related transcription factor (Runx)2, alkaline phosphatase (AP), and type I collagen (Col I) by RT-qPCR. **(F)** Representative photographs of MSCs depicted in**(E)** and stained for AP detection. Data were normalized to 1 for MSCs in SAB-containing medium. *p < 0.05 versus SAB at same time point.

We then assessed the capacity of MSCs to give rise to chondrocytes. Differentiation with SAB-containing inductive medium was confirmed by increased levels of sex-determining region Y-box9 (SOX9), aggrecan, type II collagen variant B (COL2a1Δ2) (5.7-, 7.7-, and 622-fold factor, respectively). Compared to SAB, oxidized SAB or SSc PS added in the inductive medium did not impact chondrogenic differentiation (Figure 4A). Variability between MSC-PS combinations was noticed but not related to AOPP levels or MSC samples.

Adipogenic differentiation of MSCs occurred in SAB-containing inductive medium as assessed by Oil red O staining (Figure 4B) and increased levels of all tested markers: lipoprotein lipase, peroxisome proliferator-activator receptor-γ, fatty acid binding protein 4 (11,307-, 594-, and 8.8-fold factor, respectively). When compared to SAB, MSCs cultured with SAB_400_, SAB_1000_, SAB_H2O2_, or PS tended to exhibit higher levels of differentiation markers (Figure 4C). Again, high variability between combinations of MSC-PS was observed but not related to AOPP levels or MSC samples.

Finally, in SAB-containing medium, MSCs expressed higher levels of Runt-related transcription factor (Runx)2, alkaline phosphatase, and type I collagen (Col I) (3-, 69-, and 2-fold factor, respectively) compared to proliferative conditions. Similar increase of osteogenic markers was observed when MSCs were cultured with SAB_400_, SAB_1000_, and SAB_H2O2_ although Alizarin red S and AP staining were higher (Figures 4D–F). By contrast, expression of two out of three osteogenic markers, Runx2 and Col I, were significantly enhanced by MSCs cultured with PS. These data indicated that SSc serum increased the osteogenic differentiation potential of MSCs.

### Patient Serum Reduced the Immunosuppressive Capacities of MSCs

**Figure 5:**
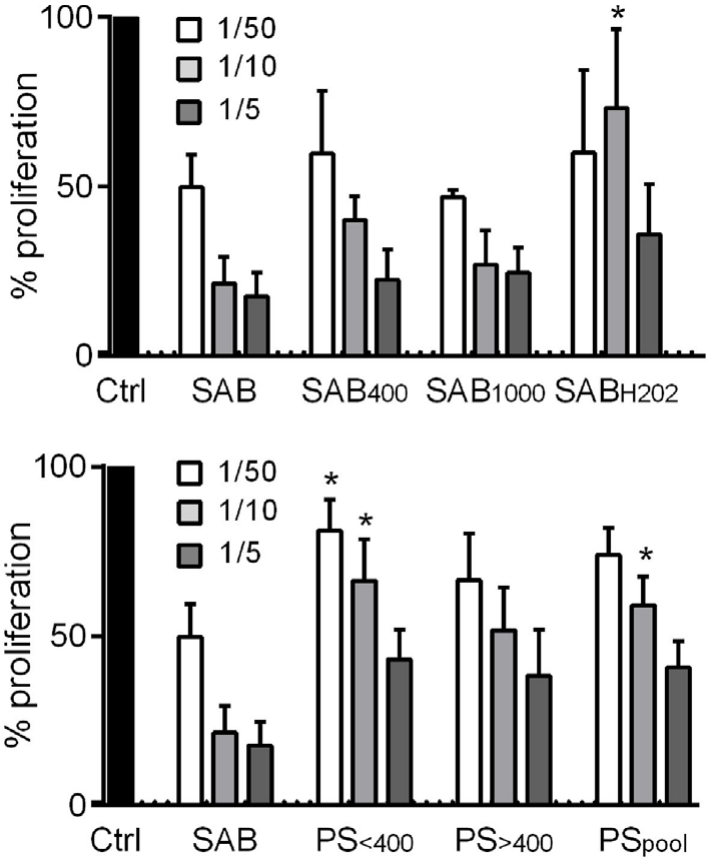
Systemic sclerosis (SSc) patient serum decreased the immunosuppressive potential of MSCs. Percentage of T lymphocyte proliferation when cultured alone (Ctrl; normalized to 100%) or with different ratios of MSCs (1/50; 1/10; 1/5 MSC/T lymphocyte ratios) that were precultured for 3 days with human serum AB (SAB) or oxidized SAB (SAB_400_, SAB_1000_, and SAB_H2O2_; *n* = 3 for each condition) or SSc patient serum. Sera from patient (PS) were divided in two groups depending on AOPP levels: <400 μmol/L (PS_<400_; *n* = 6) or >400 μmol/L (PS_>400_; *n* = 6) or pooled in a single group (PS_pool_; *n* = 12). T lymphocyte proliferation was measured with Cell Proliferation ELISA, BrdU assay (Sigma-Aldrich). Results were expressed as the percentage of proliferation ± SEM. *p < 0.05 versus SAB at same time point.

Finally, we investigated the immunosuppressive potential of MSCs when cultured with oxidized SAB or PS. A dose-dependent immunosuppressive potential was shown with MSCs precultured with SAB or oxidized SABs, except for MSCs with SAB_H2O2_ at 1 MSC/10 PBMC ratio (Figure 5). By contrast, MSCs cultured with PS exerted a lower immunosuppressive potential as observed at the 1 MSC/10 PBMC ratio. This effect was mostly attributed to the PS_<400_ group of patients. The present data highlighted a reduced immunosuppressive potential of MSCs when cultured with SSc PS.

## Discussion

Some previous studies focused on the characteristics of SSc-MSCs for their possible therapeutic use for treating patients suffering from the most severe forms of SSc (13). However, to our knowledge, this is the first study evaluating the functional capacity of heterologous MSCs obtained from non-SSc donors when exposed to the oxidative environment encountered in patients, namely SSc serum. This study aimed at investigating MSC properties in this specific context of allogeneic transplantation.

Herein, we observed that oxidative stress and AOPP levels in SSc patient serum impacted the functional properties of MSCs by reducing their proliferation and immunosuppressive potentials, while apoptosis, senescence, and differentiation potential were increased. The role of ROS and H_2_O_2_ on proliferation, self-renewal, and senescence of MSCs has already been reported (14, 15). Our results are in line with previous studies on resident SSc-MSCs, which exhibited modifications of their functions and features: early senescence with higher telomerase activity, reduced proliferative activity, and even increased expression of TGF-β-RII, leading to increased sensibility to TGF-β and excessive production of type I collagen (6, 7). SSc-associated oxidative environment reduced proliferation rate and survival of resident MSCs and therefore might impact heterologous MSCs after implantation in patients. In our conditions, proliferation rates were negatively correlated with AOPP levels. Indeed, in a perspective of MSC-based therapy in SSc, it could be tempting to propose MSC infusion in patients with low AOPP levels. Of importance, the percentage of apoptotic cells was inversely related to proliferation and significantly increased at day 10 of culture. The number of senescent MSCs also increased in contact of SSc serum at day 6, and no senescence was observed at day 10. Decreased proliferation was therefore likely associated with a first phase of senescence followed by a second phase of apoptosis. Both processes were not observed earlier, indicating that continuous exposure to oxidative stress was required to reduce MSC survival. In a therapeutic setting, the oxidative environment of SSc serum will likely not affect survival of heterologous MSCs since the half-life of MSCs in the bloodstream is less than 24 h, and the majority of cells disappeared within few days (16). Of interest, even though a study reported that coculture of SSc endothelial cells with MSCs induced the expression of myofibroblastic markers by MSCs, culture with SSc serum did not change the phenotype of MSCs (17).

We also demonstrated that PS did not increase the oxidative stress in MSCs, which can be made in relation with the upregulation of SOD2 antioxidant enzyme. This is concordant with the findings that SSc-MSCs can counteract oxidative stress by improving antioxidant defenses (18) and suggests that the antioxidative functions of MSCs were preserved under SSc oxidative environment. Other factors secreted by MSCs can play a role in their antioxidant defenses such as heme oxygenase-1 (HO-1) or glutathione-disulfide reductase (GSR). No increase of these factors was detected in our conditions (data not shown). However, the operative factors produced by MSCs may vary in different experimental conditions, likely due to different levels of ROS produced (19). This may explain the absence of upregulation of HO-1 or GSR in our settings or the production of other factors that are still to be identified. This will need to be further investigated. Interestingly, NO production by MSCs was increased following incubation with intermediate and high doses of HOCl-induced AOPPs contained in SAB, but only with intermediate doses of AOPPs contained in PS. A possible explanation could be an increased antioxidative capacity of MSCs subjected to the highest oxidative environments. However, in our conditions, both sera with low and high levels of AOPPs increased SOD2, suggesting that AOPP levels alone cannot explain this discrepancy. Indeed, PS likely conveyed both prooxidative and antioxidative components that activated the antioxidative function of MSCs. Defense against oxidative stress may also be associated to the response to DNA damage and senescence. A number of molecules are common to these processes and may be called “senescence suppressors” (20). These are promising targets for increasing cell proliferation and differentiation capacities of MSCs and also delay the onset of senescence. Treatments that could increase expression of these senescence suppressors might help MSCs maintaining or enhancing their properties and therapeutic efficacy. This is of importance for MSC-based therapy in SSc where the main effect of the treatment would be to reduce the oxidative stress contributing to the clinical manifestations of the disease.

Considering differentiation potential of MSCs, a high variability between PS was observed, while our results were quite homogenous when considering the other characteristics of MSCs, probably due to lower number of replicates. This variability was not clearly explained by individual characteristics of SSc patients (no correlation was found) or by AOPP levels. Finally, we noticed that differentiation potential of MSCs was not significantly altered, except for osteogenesis, which was increased following incubation of MSCs with PS. Previous data reported that ROS and H_2_O_2_ inhibited osteogenesis, but other reports showed that ROS increased calcification and osteogenesis (21). In addition, induction of osteogenesis in MSCs was associated with an upregulation of SOD2 and antioxidant enzymes but a decrease in ROS. In this study, when cultured in the presence of SSc sera PS, MSCs exhibited an increase of antioxidant capacity, which can be associated to osteogenesis, while the presence of ROS in sera could also participate to increase osteogenic differentiation potential. However, we cannot exclude the possibility that factors other than oxidative components and present in PS might impact MSC differentiation potential. With regard to adipogenesis, a ROS increase was associated with adipogenesis, and antioxidant enzymes such as SOD were upregulated during adipogenic differentiation (22). Several studies also reported the proadipogenic effect of ROS and H_2_O_2_ (23). In concordance with our study, some others demonstrated similar differentiation potential of SSc-MSCs and MSCs obtained from healthy donors (9, 13, 24). Scuderi et al. also reported no alteration in phenotype, differentiation, or proliferation potentials of MSCs, which were obtained from adipose tissue of six SSc patients and compared with those from healthy donors (25). Those studies and our study might suggest that slight alterations of MSC functions may be a consequence of SSc-associated oxidative environment and related to differences in patient characteristics but not to a primitive cell dysfunction. Alterations of differentiation potential might be deleterious in the context of SSc, and enhanced osteogenic differentiation of MSCs could lead to subcutaneous calcifications in those patients. However, in hands series, no relationship was established between clinically patent subcutaneous calcifications and differentiation capacities. In addition, unwanted differentiation capacity of MSCs has been rarely if ever documented in the thousands of patients who received MSCs in clinical trials while safety of MSCs is prone. To our knowledge, no ossification was reported in patients following MSC-based treatments.

Another important finding of this study is the decreased immunosuppressive function of heterologous MSCs in the presence of PS, which was not observed in the presence of HOCl-induced AOPPs containing sera. Therefore, our data suggest that other components of PS likely impact on the immunomodulatory properties of MSCs. In the study by Cipriani et al., MSCs obtained from SSc patients acquired senescence characteristics, but they maintained their immunosuppressive capacities on lymphocyte proliferation (8). Similarly, another study demonstrated similar immunosuppressive functions of SSc-MSCs compared to MSCs obtained from healthy donors (24, 26). However, MSCs from patients with other diseases associated with elevated oxidative stress, such as atherosclerosis and type 2 diabetes, also exhibited reduced ability to inhibit T-cell proliferation (27). Our findings suggest that SSc oxidative environment could exert a deleterious effect on MSC immunosuppressive functions, maybe impairing efficacy of MSC transplantation in some SSc patients. The immunosuppressive capacity of MSCs strongly contributes to their therapeutic effects in autoimmune diseases (10). These effects might be important in SSc, and we recently demonstrated that the therapeutic efficacy of MSCs in the HOCl-induced murine model of SSc was mediated through the reduction of tissue inflammation resulting in fewer macrophages and T-cell infiltrates and lower levels of pro-inflammatory cytokines (5). Indeed, even in these oxidant conditions close to the human disease, MSCs were shown to be therapeutically efficient, suggesting that MSCs adapted to the environment and preserved their functionalities.

In conclusion, we showed that in the presence of oxidative environment of PS, MSCs retained their proliferative potential, survived for at least the first 3 days of exposure, and enhanced their antioxidant capacity and osteogenic potential, whereas their immunosuppressive function was reduced. These findings were globally homogenous among PS, even if clinical presentation of our patients was heterogenous. MSCs still represent a promising therapy for SSc, independently of the source (adipose tissue or bone marrow) and histocompatibility (autologous, allogenic, and xenogenic) (12). In addition, according to the very first studies in humans, MSC-based therapy was well tolerated and seemed efficient for refractory SSc, including those with severe vascular complications such as of digital ulcers or distal limb necrosis (28). These promising results still need to be confirmed in the ongoing clinical trials and further randomized controlled studies.

## Author Contributions

Design of the study: DN, CJ, and PG. Acquisition of data: GF, CB, RG, AM, AQ, and SR. Data analysis: GF, CB, RG, AM, PG, and DN. Manuscript preparation: DN, GF, and PG. All authors reviewed the manuscript and gave final approval for the work.

## Conflict of Interest Statement

The authors declare that the research was conducted in the absence of any commercial or financial relationships that could be construed as a potential conflict of interest.

## Abbreviations

AOPP, advanced oxidation protein product; HOCl, hypochloric acid; H2O2, hydrogen peroxide; MSC, mesenchymal stromal/stem cell; PBMC, peripheral blood mononuclear cell; PS, patient serum; RNS, reactive nitric species; ROS, reactive oxygen species; NO, nitric oxide; SAB, serum AB; SSc, systemic sclerosis.
