# Supplementary material for: Serum-Mediated Oxidative Stress from Systemic Sclerosis Patients Affects Mesenchymal Stem Cell Function
Source: Front Immunol. 2017 Sep 1;8:988. doi: 10.3389/fimmu.2017.00988 (PMC5585199; doi:10.3389/fimmu.2017.00988)
Supplement: Supplementary file 1 [file table_1.pdf]

Supl Table 1: Primer sequences

Genes	Forward	Reverse
**p27**	ATAAGGAAGCGACCTGCAACCG	TTCTTGGGCGTCTGCTCCACAG
**p21**	AGGTGGACCTGGAGACTCTCAG	TCCTCTTGGAGAAGATCAGCCG
**p16**	GAAGGTCCCTCAGACATCCCC	CCCTGTAGGACCTTCGGTGAC
**SOD2**	CTGGACAAACCTCAGCCCTAAC	AACCTGAGCCTTGGACACCAAC
**TFGβRII**	TGGGCTTTCCCTGCGTCTGG	CTTGCAGTTCCCACCTGCCCA
**COL I**	CCTGGATGCCATCAAAGTCT	CGCCATACTCGAACTGGAAT
**A-Sma**	CATCGGGATGGAGTCTGCTG	AGAAGCATTTGCGGTGGACA
**Col III**	GTGAACCTGGGCAAGCTGGT	GGCCTGGTTGACCATCACTG
**TGFβ1**	GCCATGAGAAGCAGGAAAGGCCGGT	ACTGCAAGTGGACATCAACGGGTTCAC
**BCL2**	CTCGTCGCTACCGTCGTGACTTCG	CAGATGCCGGTTCAGGTACTCAGTC
**BAX**	TCAGGATGCGTCCACCAAGAAG	TGTGTCCACGGCGGCAATCATC
**RPS9**	GATTACATCCTGGGCCTGAA	ATGAAGGACGGGATGTTCAC
**COL2a1Δ2**	CAGACGCTGGTGCTGCT	TCCTGGTTGCCGGACAT
**SOX9**	AGGTGCTCAAAGGCTACGAC	GTAATCCGGGTGGTCCTTCT
**ACAN**	TCGAGGACAGCGAGGCC	TCGAGGGTGTAGCGTGTAGAGA
**LPL**	GTCCGTGGCTACCTGTCATT	TGGATCGAGGCCAGTAATTC
**PPAR-γ**	CCAGAAAGCGATTCCTTCAC	TGCAACCACTGGATCTGTTC
**FABP4**	ATGGGATGGAAAATCAACCA	GTGGAAGTGACGCCTTTCAT
